# Supplementary figures and images for: Dependency of human and murine LKB1-inactivated lung cancer on aberrant CRTC-CREB activation
Source: eLife. 2021 Jun 18;10:e66095. doi: 10.7554/eLife.66095 (PMC8238510; doi:10.7554/eLife.66095)

## Unedited gels for Figure 1B

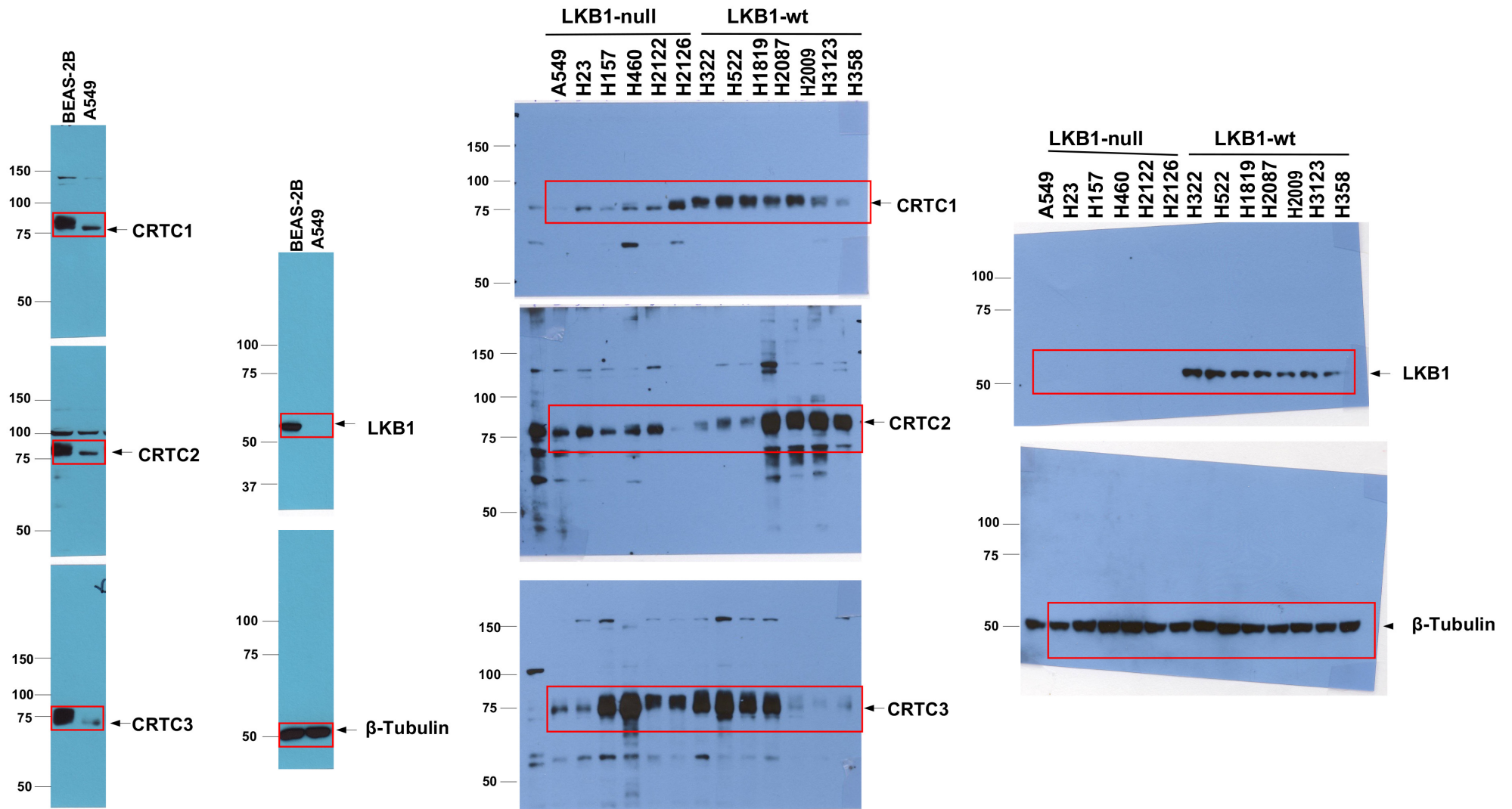

Supplement: Figure 1—source data 2. [file elife-66095-fig1-data2.pdf]

## Unedited gels for Figure 1 - figure supplement 1A

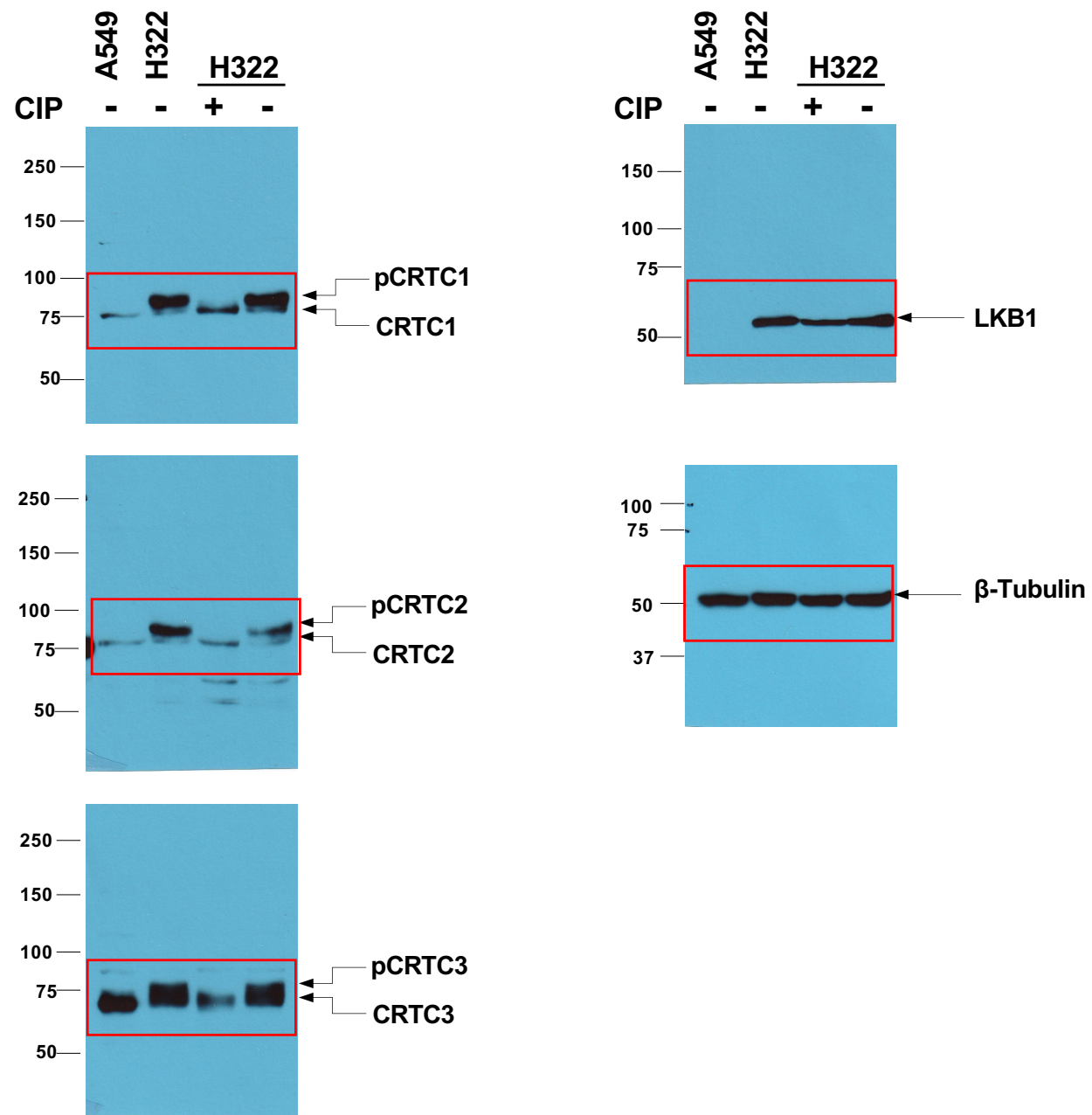

## Unedited gels for Figure 1 - figure supplement 1B:

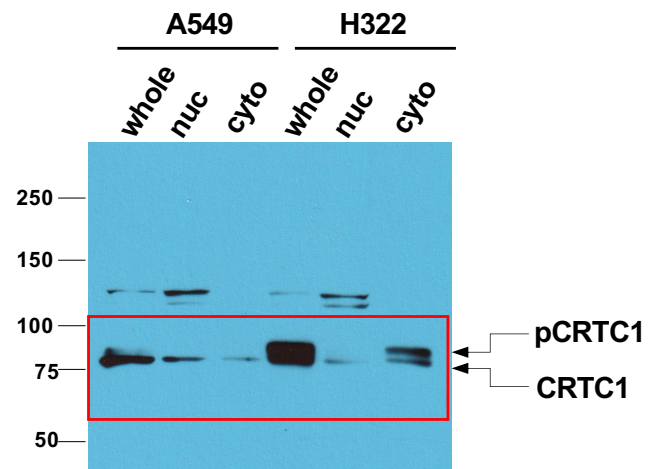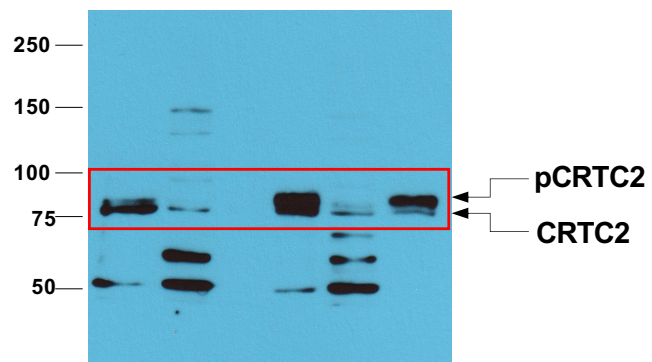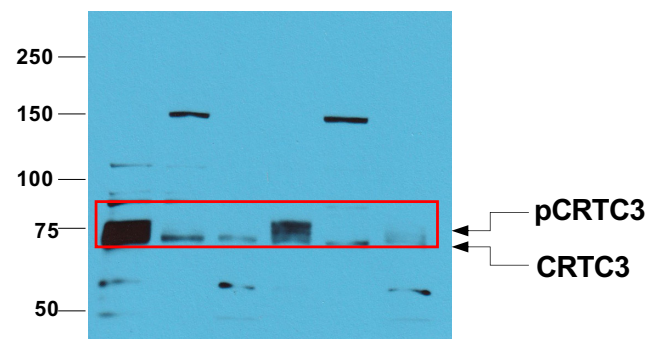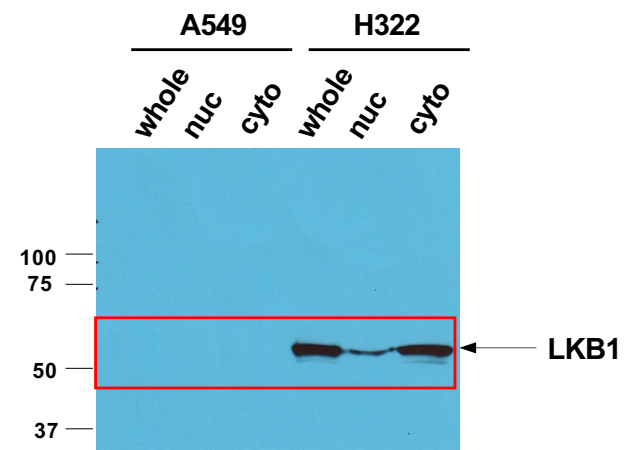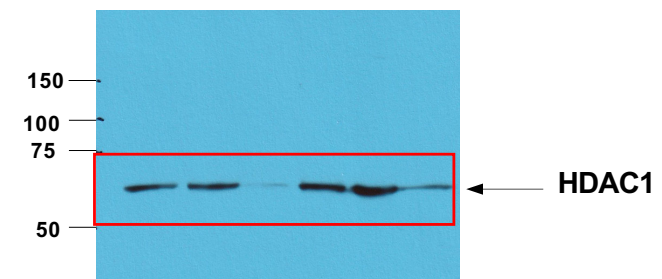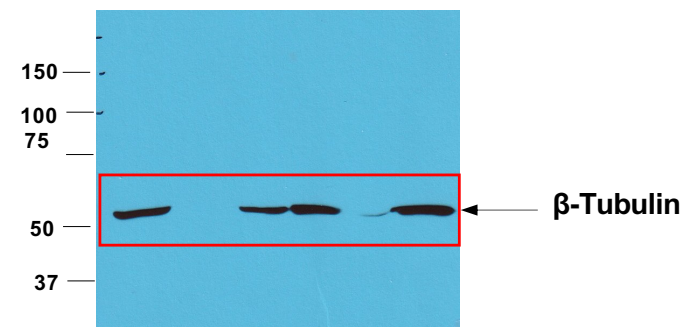

Supplement: Figure 1—figure supplement 1—source data 1. [file elife-66095-fig1-figsupp1-data1.pdf]

## Unedited gels for Figure 1 – figure supplement 2A

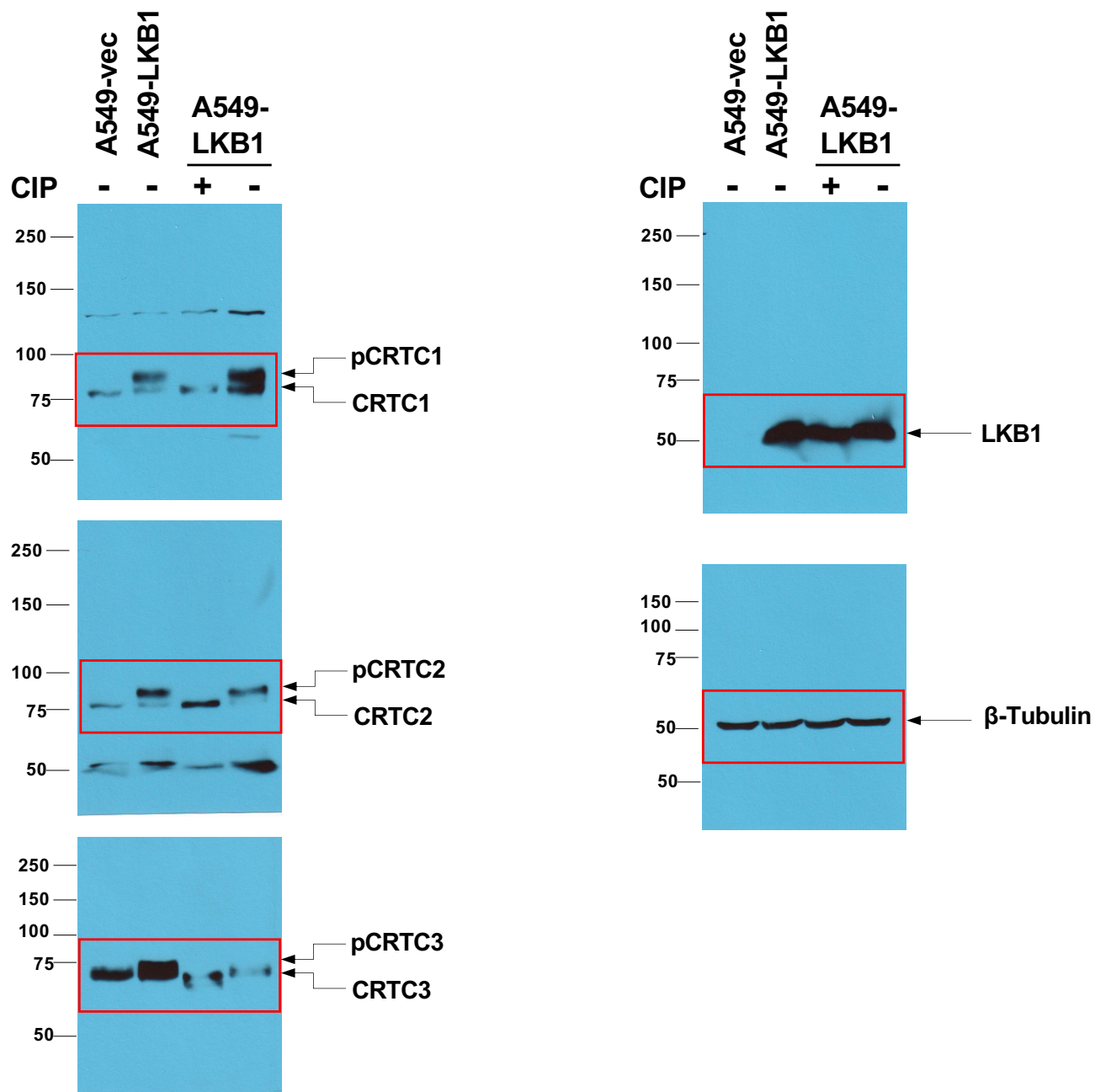

## Unedited gels for Figure 1 – figure supplement 2B

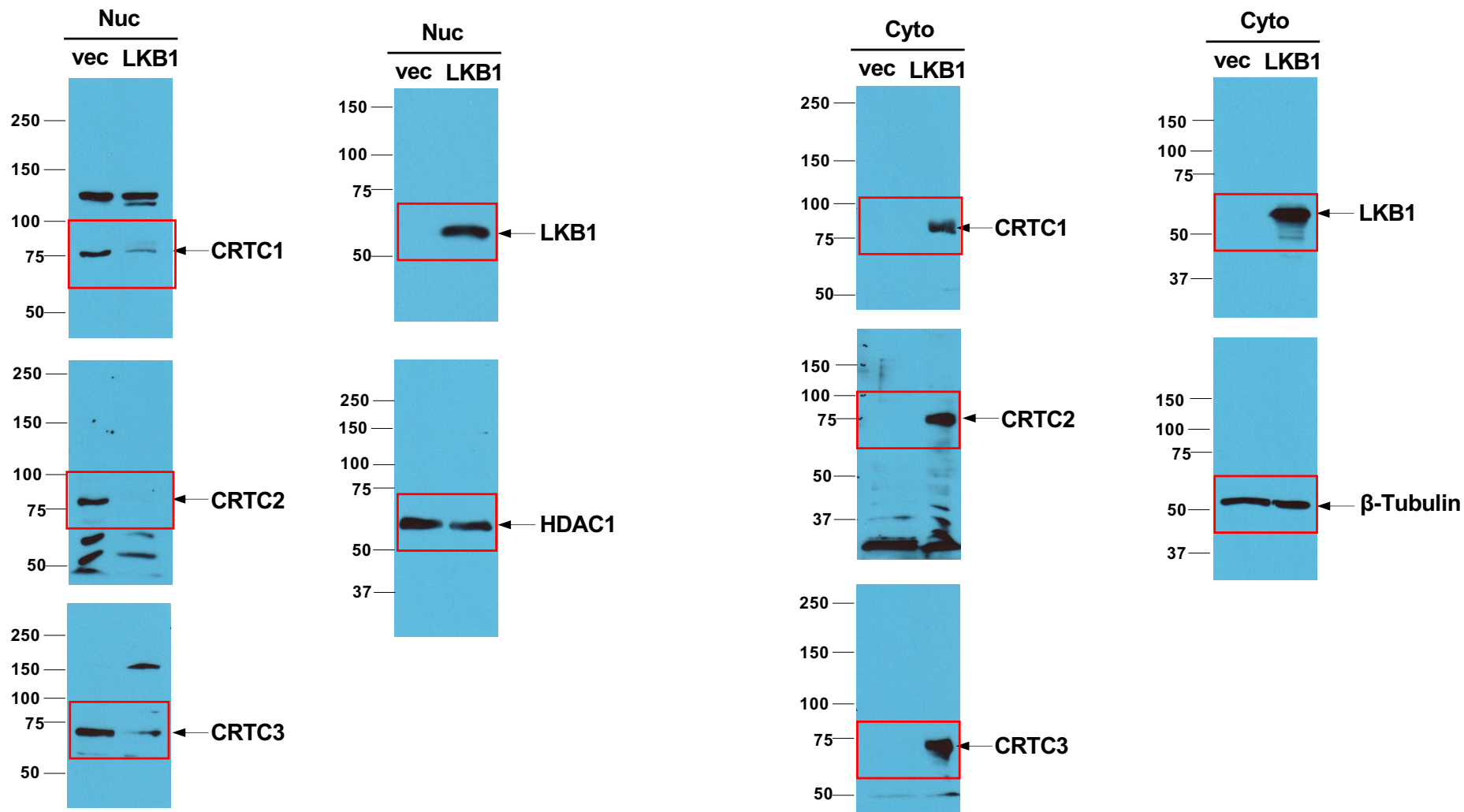

Supplement: Figure 1—figure supplement 2—source data 1. [file elife-66095-fig1-figsupp2-data1.pdf]

## Unedited gels for Figure 1 - figure supplement 3A

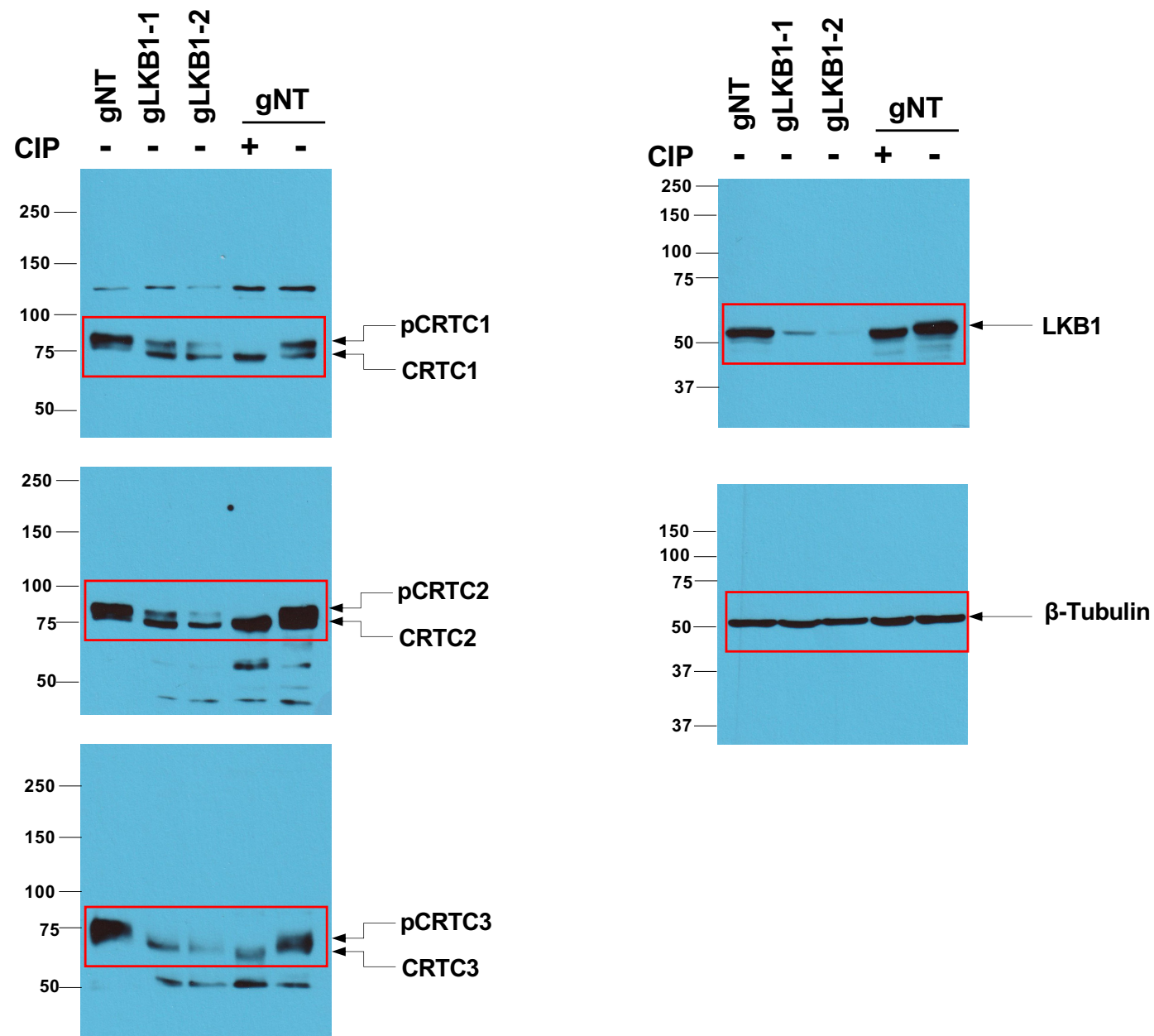

## Unedited gels for Figure 1 - figure supplement 3B

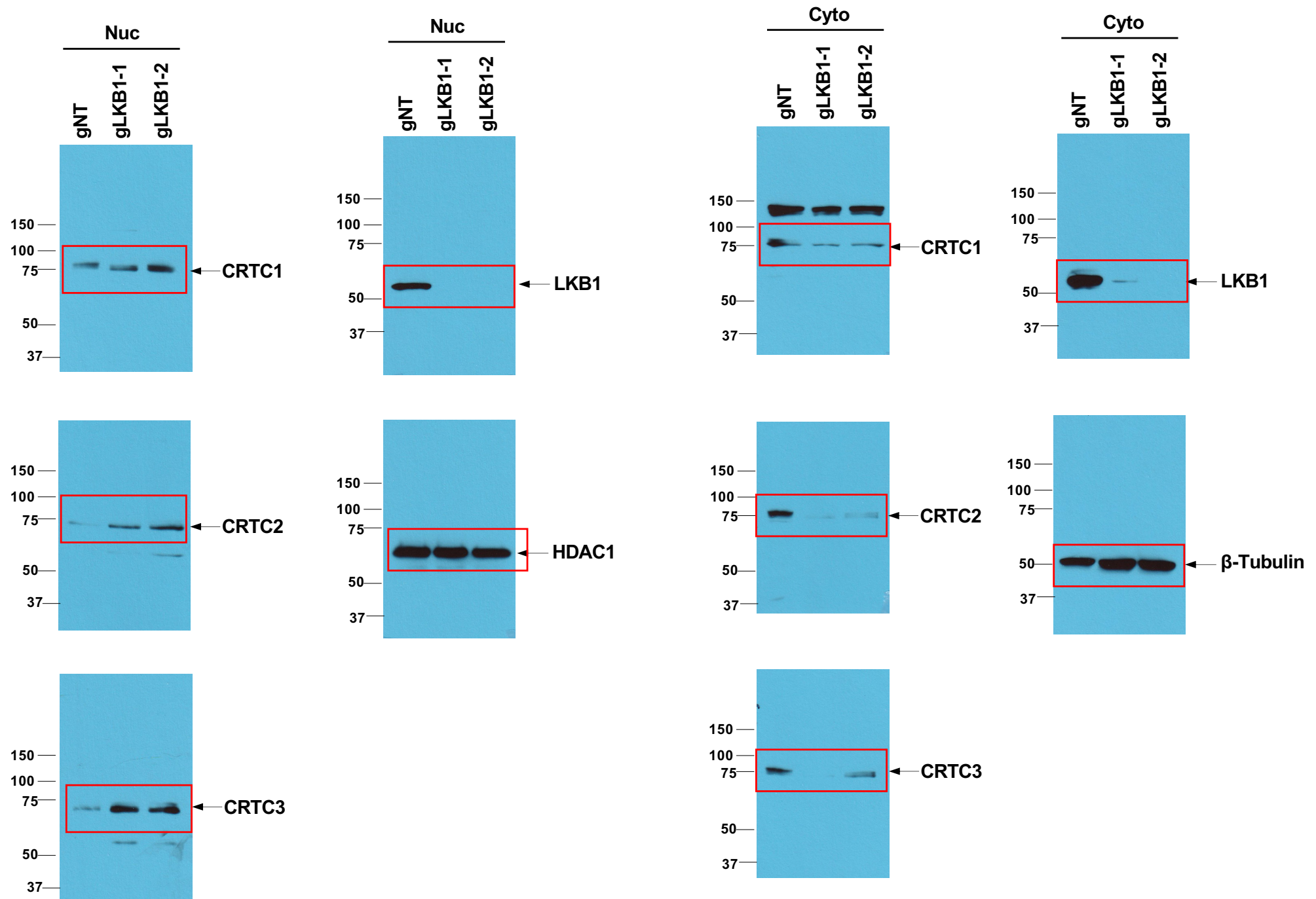

Supplement: Figure 1—figure supplement 3—source data 1. [file elife-66095-fig1-figsupp3-data1.pdf]

## Unedited gels for Figure 2A

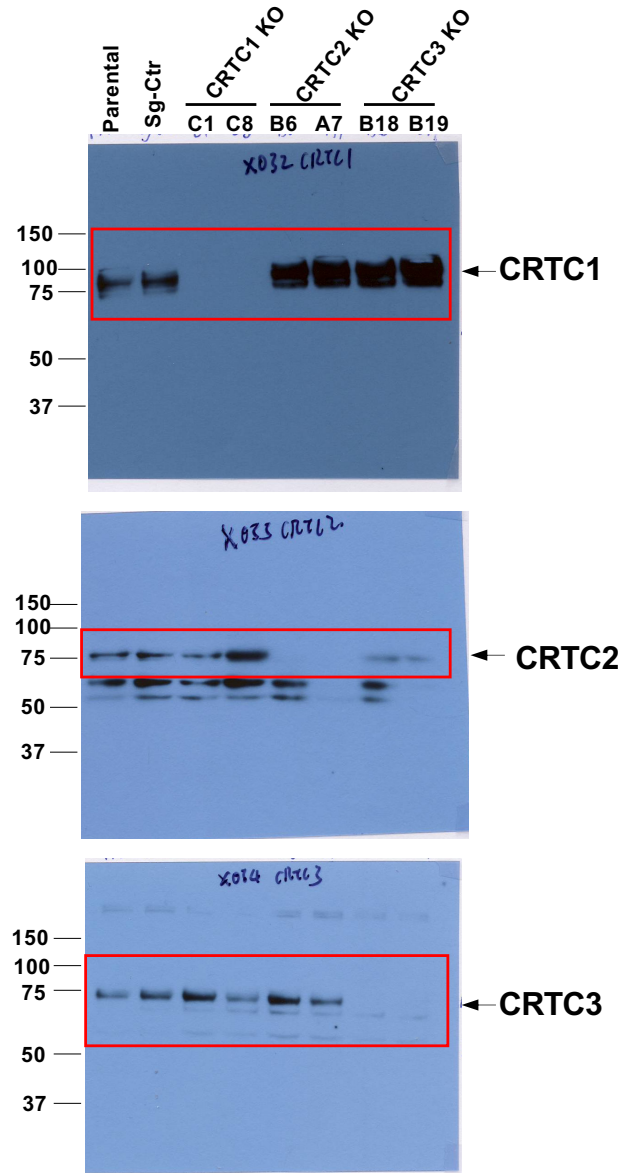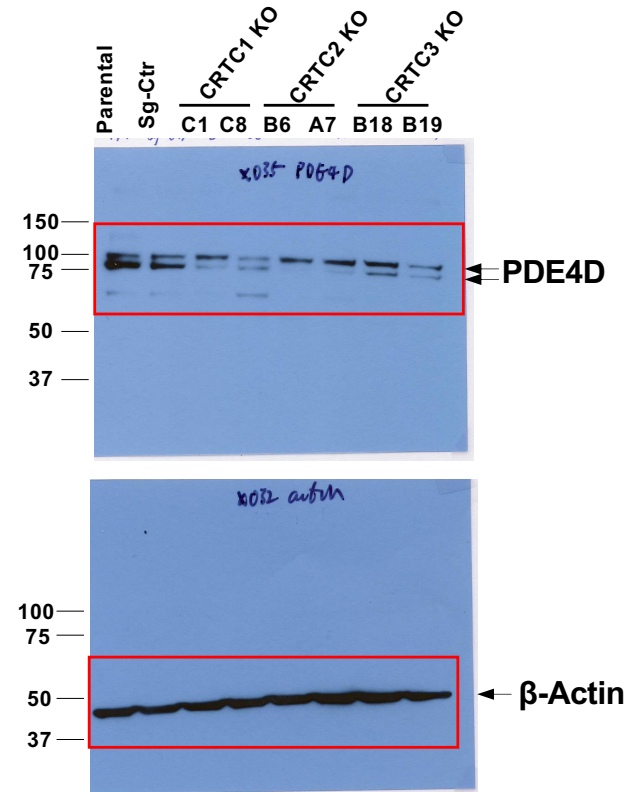

Supplement: Figure 2—source data 1. [file elife-66095-fig2-data1.pdf]

Unedited gels for Figure 3C, 3E

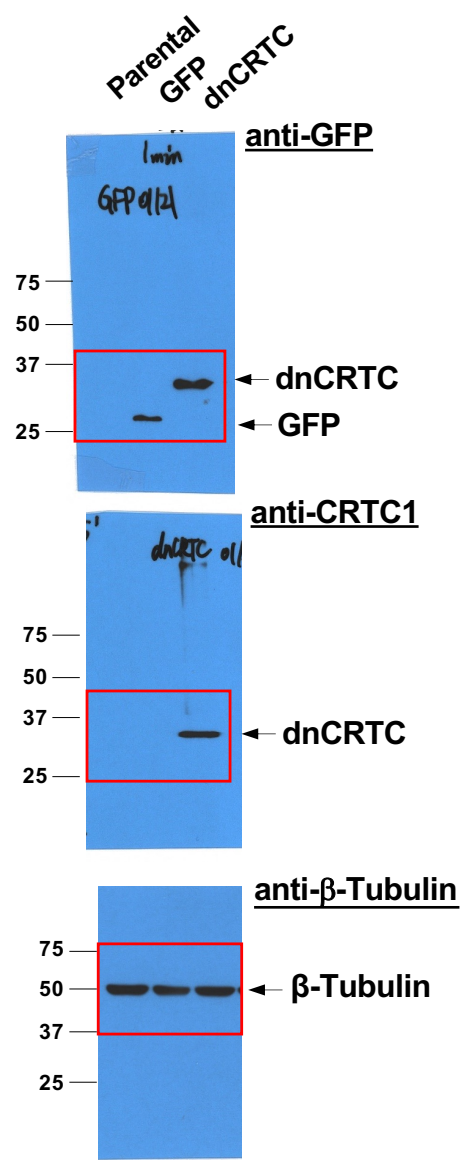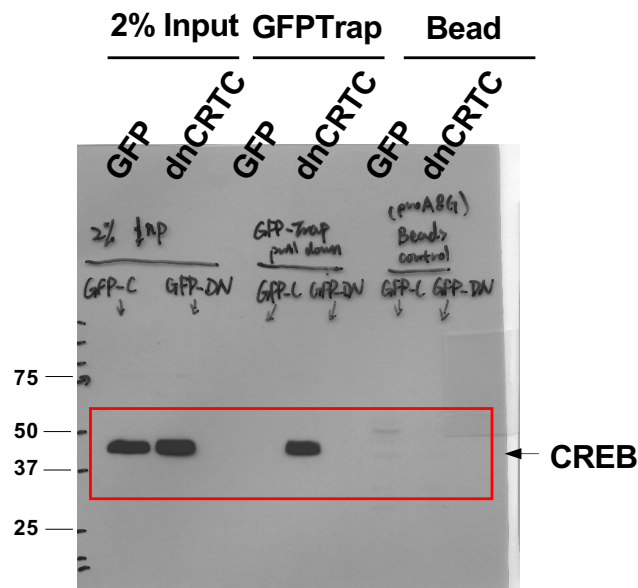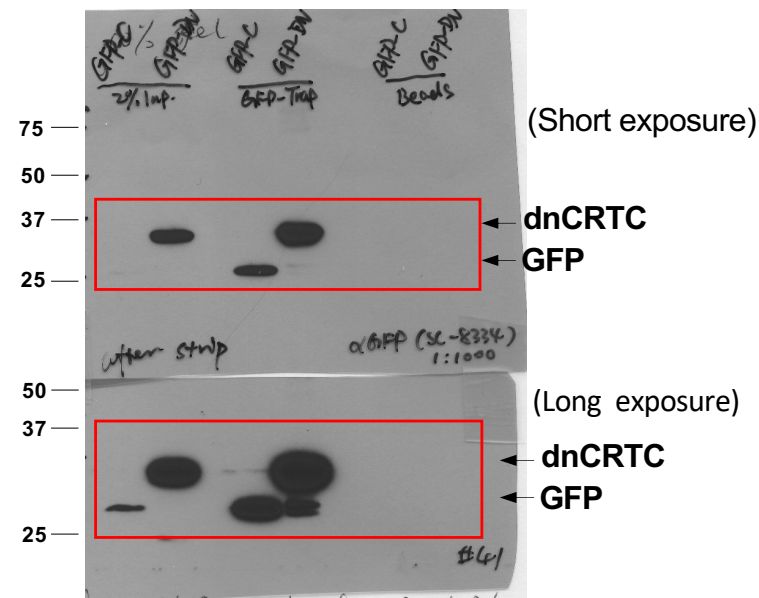

Supplement: Figure 3—source data 1. [file elife-66095-fig3-data1.pdf]

## Unedited gels for Figure 4A

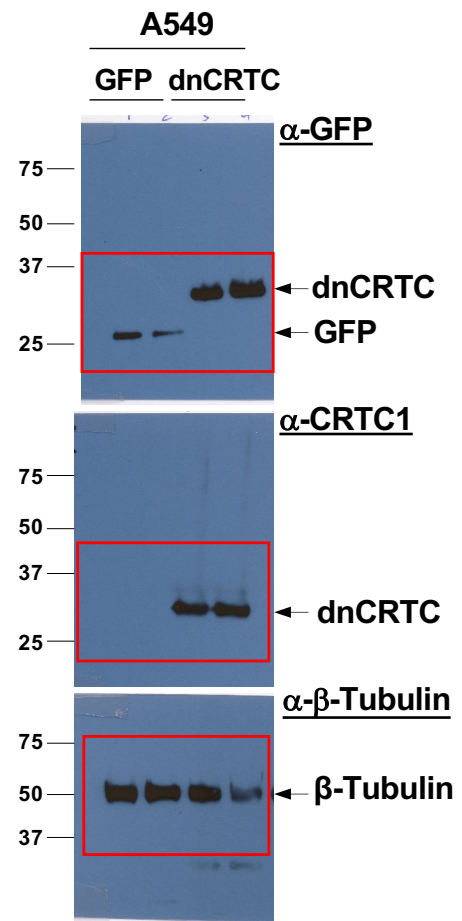

Supplement: Figure 4—source data 1. [file elife-66095-fig4-data1.pdf]

## Unedited gels for Figure 5B

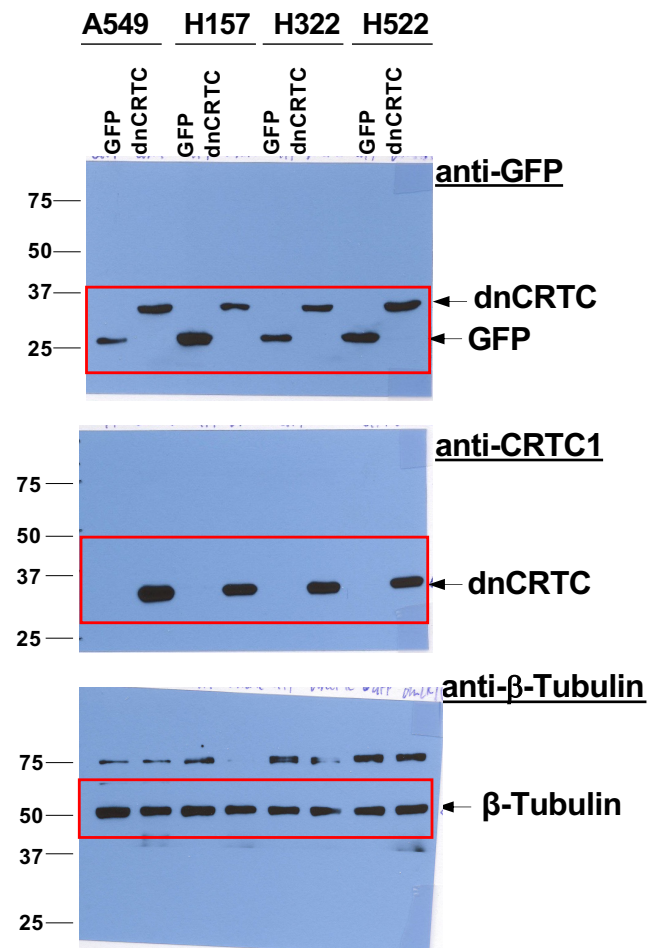

Supplement: Figure 5—source data 2. [file elife-66095-fig5-data2.pdf]

## Unedited gel for Figure 5 - figure supplement

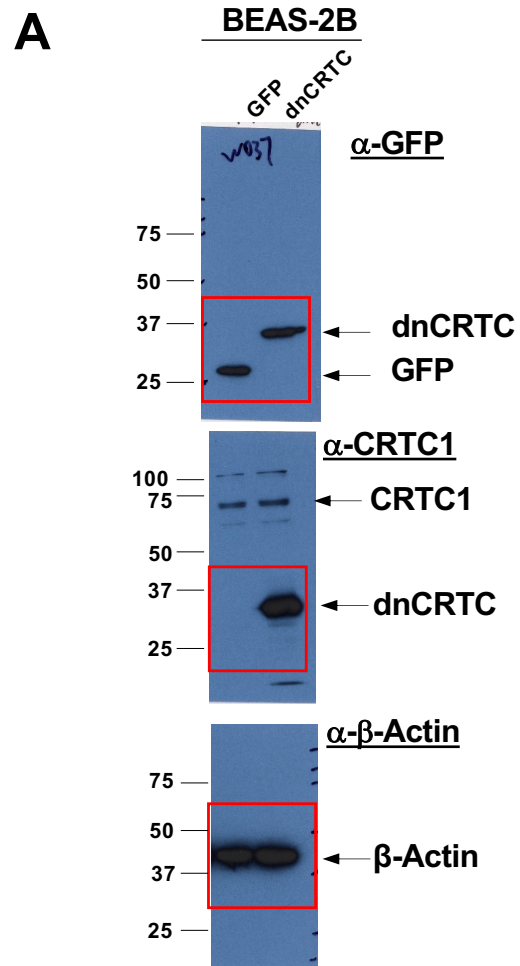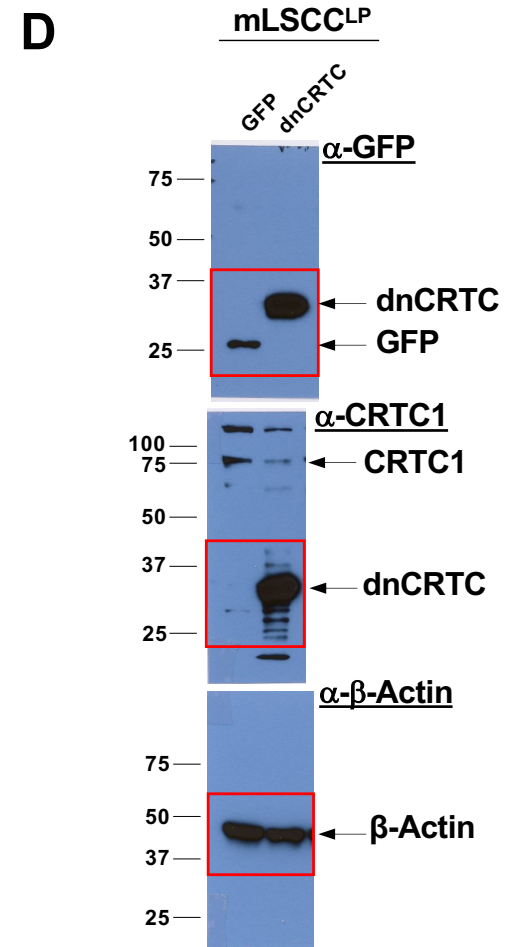

Supplement: Figure 5—figure supplement 1—source data 1. [file elife-66095-fig5-figsupp1-data1.pdf]

## Unedited gel for Figure 6 – figure supplement

**A**

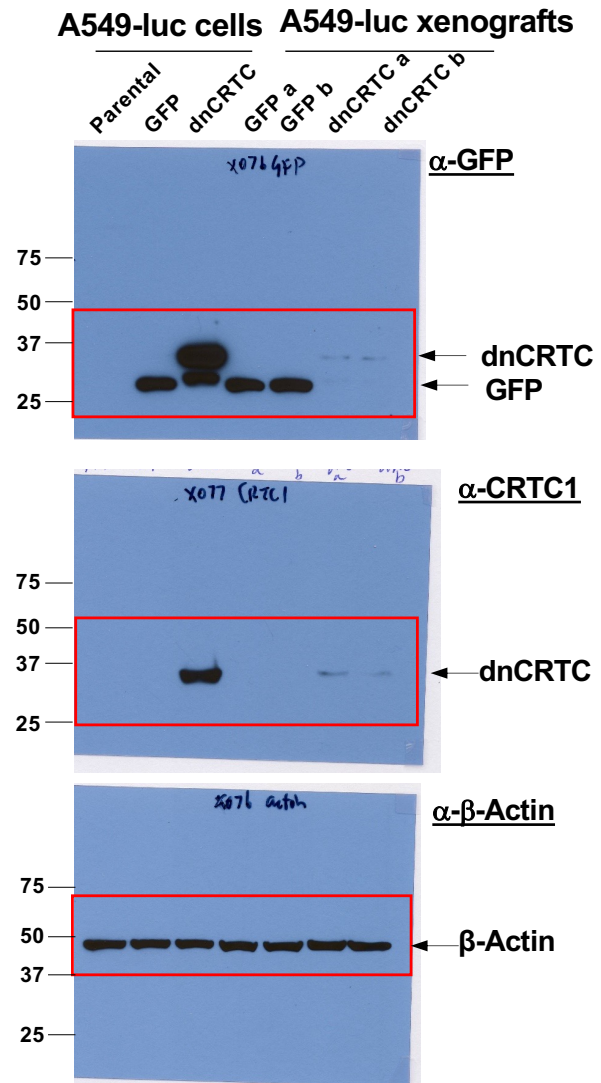

**B**

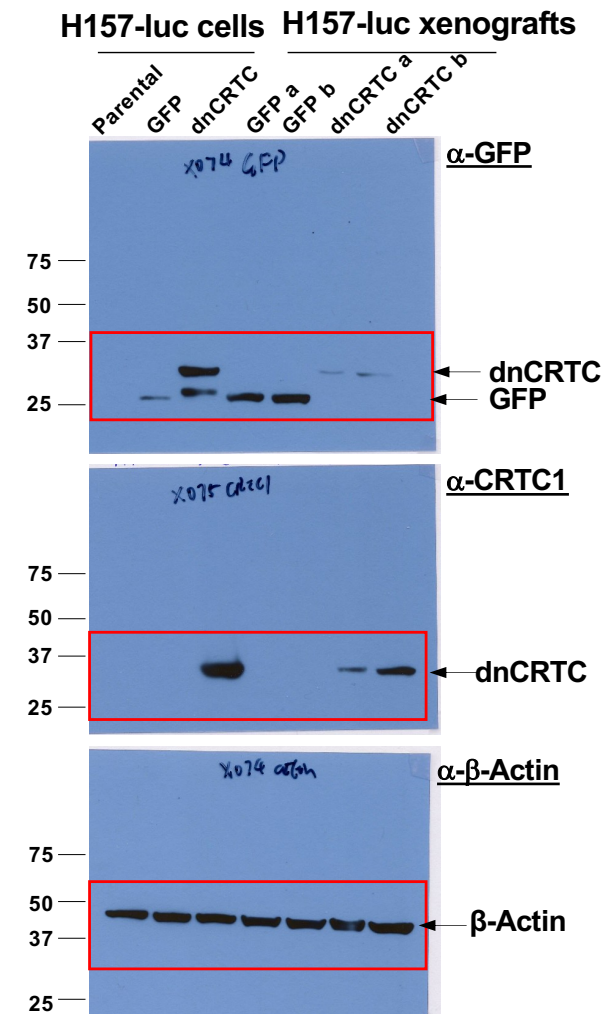

Supplement: Figure 6—figure supplement 1—source data 1. [file elife-66095-fig6-figsupp1-data1.pdf]
